# Attach importance to the individualized treatment of adult portal hypertension based on etiology and pathogenesis: A review

**DOI:** 10.1097/MD.0000000000033527

**Published:** 2023-04-21

**Authors:** Ming-ke Li, Lan-qing Ma

**Affiliations:** a Digestive Department, The First Affiliated Hospital, Yunnan Institute of Digestive Disease, Yunnan Clinical Research Center for Digestive Diseases, Kunming Medical University, Kunming, China.

**Keywords:** etiology, individualized treatment, pathogenesis, portal hypertension

## Abstract

There are many factors that can cause portal hypertension and secondary symptoms such as ascites, splenomegaly, and variceal hemorrhage, can seriously affect patients’ quality of life and even threaten their lives. In this paper, we summarize various causes of portal hypertension based on etiology and pathogenesis and give individualized treatment strategies in order to remind clinicians to pay attention to the identification of different causes and select corresponding treatment, so that patients are provided with the optimal treatment strategies and benefit from them.

## 1. Introduction

In 2021, experts from China, the United Kingdom, and the United States jointly published a review in *Hepatology International* “A Community of Portal Hypertension,” which once again aroused our attention to the clinical diagnosis, treatment, and scientific research of portal hypertension (PH) and emphasized the joint development of multiple disciplines and regions.^[1]^ PH is a clinical syndrome caused by a pathological increase in portal pressure (PP) that itself can be due to various problems, such as liver cirrhosis, which is the most common cause.^[2]^ Other rarer causes of PH include portal vein thrombosis, arteriovenous shunt (AVS) or splenomegaly, and idiopathic non-cirrhotic portal hypertension (INCPH). Ascites, gastroesophageal varices (GEVs) and splenomegaly are the main manifestations of PH. Variceal hemorrhage (VH) occurs at a rate of about 5% to 15% in PH patients and can dramatically affect their quality of life and even threaten their lives.^[3]^ The treatments of PH include both etiological and symptomatic treatments that primarily involve drugs, as well as endoscopy, intervention, and surgery. In the face of complex conditions, how to choose effective treatment strategies among many schemes to bring the most benefit to patients is an important question for clinical practitioners.

## 2. Diagnosis of PH

Currently, hepatic venous pressure gradient (HVPG) is widely used to evaluate the changes in PP and is also the best method for the diagnosis of PH.^[4]^ Normal PP is defined by an HVPG ≤ 5 mm Hg. Subclinical PH, also known as mild PH in China, is defined by an HVPG > 5 but < 10 mm Hg. An HVPG ≥ 10 mm Hg is known as clinically significant portal hypertension (CSPH).^[2,5]^ In addition, HVPG is also used to assess the risk level of patients with PH and to predict the risk of VH. According to their HVPG, patients with acute VH can be divided into a high-risk group (HVPG ≥ 20 mm Hg) and a low-risk group (HVPG < 20 mm Hg). HVPG measurement not only requires specific expertise but is invasive and relatively expensive as well, so it has not been available in all treatment centers. To address this difficulty, a prospective study published in *Gastrointest Endosc* stated that EUS-guided portal pressure gradient (EUS-PPG) was performed using a 22-gauge FNA needle and a central venous pressure measurement monitor. Compared with transjugular approach, the Pearson correlation coefficient of 0.923 (*P* < .001). As a safe and accurate method, EUS-PPG is expected to be a supplementary method for the detection of HVPG.^[6]^ At the same time, German researchers have found that MRI imaging index – splenic extracellular volume fraction significantly correlated with PH (*P* < .001) and direct HVPG (*P* = .003), which may play a role in noninvasive quantitative analysis of portal hypertension in the future.^[7]^ Now, we can diagnose CSPH according to the following methods. First, the presence of portosystemic collateral circulation on ultrasound, CT, or MRI is specific for CSPH. Second, GEVs that can be seen under endoscopy also indicate CSPH.^[3]^ Thirdly, the combination of liver stiffness measurement (LSM) and platelets count also has an indicative effect on CSPH: LSM ≤ 15 kPa and PLT ≥ 150 × 10^9^/L can be used to exclude CSPH. While LSM ≥ 25 kPa can indicate CSPH.^[8]^

## 3. Pathogenesis of esophageal and gastric varices in PH

The mechanism of PH consists of 2 aspects: the increase in vascular resistance and blood flow of the portal vein system, and these 2 factors interact, especially in the progression of liver cirrhosis. PP initially increases attributes of structural components, like the formation of regenerative nodules and microthrombi as a consequence of increased intrahepatic resistance. Furthermore, the decrease in nitric oxide bioavailability that results in endothelial dysfunction is another reason for increased intrahepatic resistance. However, increased production of nitric oxide leads to dilation of circulatory system and visceral vessels, leading to activation of neurohumoral and vasoconstrictive systems, which then aggravate sodium and water retention, increasing blood volume and cardiac output. That is, a hyperdynamic circulatory state that ultimately increases PP and portal venous inflow occurs. In addition, systemic neurohumoral activation and vasoconstriction may gradually contribute to intrahepatic vasoconstriction.^[9–11]^ In recent years, researchers have gradually observed that vascular endothelial growth factor and platelet-derived growth factor also play important roles in the formation of PH.^[12]^ In other diseases, such as Budd-Chiari syndrome (BCS), pancreatic portal hypertension (PPH), and vena cava thrombosis are often due to vascular obstruction. Besides, hepatic AVS, megaspleen, and lymphoma, can also contribute to excessive blood flow into the portal vein, eventually leading to PH.

## 4. Clinical features of GEVs

Under endoscopy, varicose veins with diameters <5 mm, or varicose veins in a straight line are defined as “mild.” Varicose veins that are serpentine, tortuous and uplifted, or linear combined with red signs are deemed “moderate.” If the varicose veins are beaded, nodular, or tumor-like, regardless of other factors such as red signs, these are “severe” varicose veins. Besides, at present, the Sarin classification is particularly useful to describe the distribution of varices in the esophagus and stomach found by endoscopy.^[13]^ There are 4 common categories according to this classification. Type 1 GEVs show continuous esophageal and gastric varices that extend along the lesser curvature of the stomach to 2 to 5 cm below the gastroesophageal junction, which is considered to be the farthest extension of esophageal veins. Type 2 GEVs is when the varices extend along the great curvature of the fundus of the stomach and beyond the gastroesophageal junction. However, isolated gastric fundal varices are defined as Type 1 isolated gastric varices. Type 2 isolated gastric varices are ectopic gastric varices in the gastric fundus, antrum, or around the pylorus, which is very rare. Fully understanding the formation mechanisms of PH and the anatomical characteristics of vascular routing is particularly important for the choice of follow-up treatment.

## 5. Etiological treatment under different mechanisms

### 5.1. Cirrhosis

#### 5.1.1. Medical treatment.

For the etiology of liver cirrhosis, such as hepatitis B or hepatitis C virus infection, studies have shown that after effective antiviral treatment, a considerable number of patients achieved liver histological reversal. Moreover, esophageal varices were improved, and PH was even reduced.^[14]^ nonselective β-blockers (NSBBs) are one of the most well-known drugs for GEVs in PH and are used for the primary prevention of VH. Research has shown that in patients with liver cirrhosis, for those without GEVs, NSBBs have no significant advantage in the prevention of late complications and mortality compared to the controls, however, and are therefore not recommended for this kind of patient. Patients with mild varicose veins usually need only endoscopic follow-up without preventive treatment, unless they are at great risk of bleeding, but for patients with moderate and severe varicose veins, studies have found that active preventive treatment can significantly reduce the risk of first bleeding and mortality. Compared to endoscopic variceal ligation (EVL), NSBBs have the same preventive effect and can reduce PP by reducing cardiac output and contracting visceral vessels. Moreover, NSBBs can reduce the occurrence of bacterial translocation, ascites, and spontaneous bacterial peritonitis.^[15]^ For patients with VH, once bleeding occurs, it must be treated urgently. Vasoactive drugs are most often used clinically and are recommended to be used as soon as possible before endoscopic treatment.^[16]^ These drugs primarily reduce PP by inducing visceral vasoconstriction. Somatostatin and its analogs, such as octreotide, can effectively reduce about 15% of HVPG and effectively prevent rebleeding and are widely used in clinical due to their rare side effects.^[17]^ In addition, vasopressin and its analogs, such as terlipressin, are the strongest vasoconstrictors available and have strong hemostatic effects but are rarely used due to their high incidence of cardio-cerebrovascular complications. In addition, recent studies have found that statins may significantly decrease fibrogenesis, adjust intrahepatic endothelial dysfunction, reduce PP, and improve hepatic perfusion and function.^[18]^ More clinical experiments are urgently needed to validate these findings, however.

#### 5.1.2. Endoscopic therapy.

Endoscopic therapy includes EVL, endoscopic injection sclerosis (EIS), and tissue adhesives. EVL and NSBBs are the primary prevention schemes for GEVs, but they are not recommended for use at the same time. The reason for this is that the combination of EVL and NSBBs does not enhance the curative effect, but the incidence of adverse events becomes much higher.^[19]^ The combination of EVL and drug treatment (as mentioned above) is the first choice for acute VH, for their better curative effect and fewer complications.^[3]^ EVL by itself can be repeated once after 2 to 4 weeks until the varices disappear, and EIS can be used for acute bleeding of GEVs. Polylauryl alcohol is the most commonly used hardener in clinic and is usually repeated 1 to 2 weeks after the first injection for 3 to 5 times until the varicose veins disappear. A recent nationwide cohort study has indicated that EIS and EVL are both effective ways for curing acute esophageal variceal bleeding. There was no significant difference in the incidence of in-hospital mortality, early rebleeding, need for intensive care unit, and length of hospital stay of cirrhotic patients with acute esophageal variceal bleeding who received emergency EVL or EIS.^[20]^ For isolated gastric variceal bleeding, a suitable method for primary and secondary prevention is endoscopic tissue adhesive injection with common tissue adhesives such as cyanoacrylate and fibrin glue. In order to reduce the risk of ectopic embolism (such as mesenteric vein and pulmonary vein embolism), which can be the most common complication of these injections, tissue adhesives need to be injected quickly and evenly, removing the needle immediately. EUS guided endoscopic therapy has also made new discoveries: The injection of cyanoacrylate glue injection or the placement of micro-coils with synthetic fiber to promote clot formation guided by EUS, which can not only treat active bleeding from gastric varices, but also in primary or secondary prevention of recurrent bleeding from gastric varices.^[21]^

#### 5.1.3. Interventional therapy.

When acute VH cannot be controlled by drug or endoscopic treatment, or when there are patients at high-risk of treatment failure, such as those with CTP-C or CTP-B with active bleeding, and when VH occurs in patients with end-stage liver disease before liver transplantation, implementing transjugular intrahepatic portosystemic shunts (TIPS) at an early stage to help rapidly reduce PP, within 72 hours, preferably within 24 hours, is strongly recommended.^[22]^ This has the advantages of smaller incidence of trauma, lower incidence of complications, and a more than 90% effective hemostasis rate.^[4]^ Suggestions from the Japanese Society of Gastroenterology and the Japan Society of Hepatology have shown that balloon-occluded retrograde transvenous obliteration can be as useful for gastric varices as a prophylactic/preventive treatment. However, more evidence may be needed to support this.^[23]^ In the near future, with the improvement of EUS-guided intrahepatic portosystemic shunt, it can also become another effective method for the treatment of PH.^[21]^

#### 5.1.4. Surgical treatment.

About 20% of PH patients have uncontrollable bleeding, or rebleeding within 24 hours after initial bleeding has stopped. Patients who do not improve after standardized medical treatment and those who have surgical indications should undergo surgical treatment. Generally, devascularization or surgical shunt can be considered.^[24,25]^ However, liver transplantation is the only way to cure cirrhosis and PH.

### 5.2. Pancreatic portal hypertension

PPH, which is typically secondary to various pancreatic diseases such as tumors, pancreatic duct stones, or chronic pancreatitis, accounts for around 5% of extrahepatic PH.^[26]^ The anatomical relationship between the pancreas and splenic vein largely determines the pathogenesis and pathological characteristics of PPH. Pancreatic lesions can compress the splenic vein and cause its obstruction or occlusion. Moreover, patients with pancreatitis have higher risk of splenic vein thrombosis. Both of these factors can block the flow of blood from the splenic vein to the portal vein. During PPH, the left gastroepiploic vein, the posterior gastric vein, and the short gastric vein are the most important shunt veins.^[27]^ When splenic vein embolism occurs, the blood from the splenic vein can flow back to the superior mesenteric vein through the pathway from left gastroepiploic vein to right gastroepiploic vein. Meanwhile, blood flow changes towards to the gastric fundus and gastric body through the posterior gastric vein and the short gastric vein. Gradually, varicose veins appear and become aggravated. These veins are usually characterized by obvious reticular varicose vessels in the splenic hilum area and the greater curvature of gastric body and extend from the serosa layer of the gastric body to the submucosa. Compared to VH in liver cirrhosis, the incidence of bleeding in PPH is significantly lower, at about 10% to 20%. Researchers have speculated that there are 2 shunting pathways that together result in smaller blood flow through the gastric fundus and in lower pressure of gastric fundus varices.

One of the key steps in PPH treatment is the active treatment of primary pancreatic diseases before bleeding in order to alleviate PH or hypersplenism and avoid later surgical treatment. The most effective method for PPH or later VH is splenectomy, which can decrease collateral circulation blood flow, reduce varicose vein pressure, improve congestion in the gastrosplenic area, control the blood flow of the gastric and esophageal vein, cut off isolated gastric fundus varices, and help mitigate the possibility of rebleeding.^[26]^ However, researchers widely believe that prophylactic resection is not suitable for patients who do not suffer from bleeding.

Another important treatment scheme is shunt surgery. Theoretically, shunt surgery, such as coronary-caval shunt, can be performed for patients with PPH, but due to the complexity of vascular anatomy and splenic vein embolism caused by pancreatic lesions, as well as the existence of hypersplenism after shunt, this method is generally not the first choice for the treatment of PPH in practice.^[28]^ Partial embolization of the splenic artery, an interventional treatment method, has also been shown to provide a good therapeutic effect. Here, metal coils are inserted into the splenic artery to reduce blood perfusion to the splenic parenchyma.^[29]^ Also, some studies have tried percutaneous splenic phleboplasty in the treatment of PPH to restore normal blood flow of splenic portal vein and have achieved good short-term efficacy.^[30]^ However, in view of the complexity of splenic vein branch openings and the lack of long-term, multi-center systematic research with large samples, the clinical validation of this method remains to be complete. The treatment of PPH is quite different from that of intrahepatic PH caused by liver cirrhosis, so TIPS seems to be ineffective.^[31]^ For patients with acute bleeding who cannot overcome the primary disease of their pancreas or tolerate surgery, EVL or EIS might be efficacious choices.^[28]^

### 5.3. Budd-Chiari syndrome

Currently, BCS is broadly defined as the clinical syndrome of portal and/or inferior vena cava (IVC) hypertension caused by obstruction of hepatic venous outflow located from the small hepatic venules up to the entrance of the IVC into the right atrium. Due to the complex etiology of BCS, appropriate treatment schemes need to be selected according to its specific different etiology, pathological types, classification and clinical manifestations.^[32]^

According to the location and nature of lesions, we can choose different methods to treat BCS. If it is complicated with venous thrombosis, interventional or surgical treatment combined with thrombolytic therapy are needed simultaneously. Balloon dilatation is the first choice for membranous obstructive lesions usually located in the hepatic vein/accessory hepatic vein or the IVC. Obstruction of the hepatic vein or IVC greater than 10 mm is called segmental occlusion of the hepatic vein or IVC. This type of interventional therapy usually requires the implantation of intravascular stents after balloon dilatation. Segmental obstruction of the IVC, involved the occlusion of left, middle, or right hepatic veins at the same time, most of which are compensated by thick accessory hepatic veins. This is a mixed obstruction from its anatomical definition, but the thick accessory hepatic veins and their communicating branches play a role in the compensation. During interventional therapy, only the occluded IVC needs to be treated, without dealing with occluded hepatic veins. Extensive obstruction of the hepatic vein, including occlusion of the entire process or severe obstruction of multiple branches of the hepatic vein, usually leads to typically poor prognoses. TIPS is recommended. Additionally, mixed lesions with obstruction of both the hepatic vein and IVC require simultaneous interventional treatment.^[33,34]^ When TIPS cannot be performed due to abnormal and/or distorted anatomy, the only nonsurgical approaches to treat severe PH consist of the creation of an intrahepatic portocaval shunt using the percutaneous (direct intrahepatic portocaval shunt) or transjugular routes (transjugular transcaval intrahepatic portosystemic shunt). These procedures have been rapidly adopted in patients with BCS in recent years.^[35]^

In addition to interventional therapy, surgical treatment is also widely used in the treatment of BCS and includes radical correction, shunt, devascularization, and liver transplantation. Shunt such as meso-atrail shunt, can be performed in both hepatic vein occlusion and long-range occlusion or obstruction of the IVC. For patients with localized obstruction or occlusion of the IVC, or severe portal and IVC hypertension after balloon dilatation and/or stent implantation failure, meso-cavo-atrail shunt can be used. As for patients with obstruction of the hepatic vein and/or a failure of intrahepatic portosystemic shunt but an unobstructed IVC, meso-cavo shunt might be a suitable choice. Splenorenal shunt is feasible in patients with PH caused by hepatic vein obstruction but an unobstructed IVC and occlusion of the superior mesenteric vein. Devascularization can be performed on patients with localized obstruction of the IVC with secondary thrombosis, failure of balloon dilatation and/or stent implantation, or meso-atrail or meso-cavo shunt for localized obstruction of the IVC, and direct radical resection can be used to achieve centripetal blood reflux of the hepatic vein. For patients with upper gastrointestinal bleeding caused by PH whose extent of liver function damage is CTP-A or CTP-B, pericardial devascularization is feasible and can control VH both quickly and accurately. The significant increase in portal pressure after this operation ensures increased blood flow into the hepatic portal vein, which is conducive to hepatocyte regeneration and liver function improvement. The short-term and long-term effects have also been shown to be satisfactory, and the long-term rebleeding rate can be reduced to as little as 10%. Finally, liver transplantation is suitable for patients with end-stage BCS in order try to maximize a patient’s life.^[32]^

### 5.4. Idiopathic noncirrhotic portal hypertension

INCPH is a rare disease characterized by PH without cirrhosis, liver disease of other causes, or visceral vein thrombosis.^[36]^ In general, the potential pathogenesis of INCPH can be divided into 5 categories: immune-based diseases, chronic infection, exposure to drugs or toxins, genetic diseases, and a hypercoagulable state.^[37]^ Diagnosis of INCPH relies on exclusion diagnosis, including: clinical findings of symptoms or signs related to PH; eliminating the possibility of chronic hepatitis and liver cirrhosis; exclusion of portal vein thrombosis, Budd-Chiari syndrome, congenital liver fibrosis, and other causes of noncirrhotic PH; and histological manifestations of INCPH.^[38]^

According to the BavenoVI consensus, NSBBs, vasoconstrictor drugs, and endoscopic treatment principles applicable to VH in liver cirrhosis are also suitable for patients with VH in INCPH, and EVL and EIS are equally effective for variceal eradication.^[39]^ For patients with ineffective drug and endoscopic treatment or repeated bleeding, TIPS should be performed as early as possible, and partial splenic embolization can be considered in patients with hypersplenism. Researchers suggested that the rate of splenic embolization should not exceed 70% in order to reduce complications such as post embolization syndrome, abscess, and bleeding.^[40]^ In INCPH with portal vein thrombosis, the use of anticoagulant drugs has been shown to recanalize the thrombus in half of patients.^[41]^ Although there is a lack of control group testing and overall consensus, considering the risk of VH, it is still suggested that anticoagulant treatment be started when there is a potential prethrombotic state in INCPH. About 6% of patients with INCPH may need liver transplantation.^[42]^

### 5.5. Formation of hepatic arteriovenous shunts

An abnormal shortcut channel between the artery and the vein without passing through the capillary network is referred to as an AVS. Hepatic AVS includes hepatic arterial hepatic venous shunt, hepatic arterial portal venous shunt, and mixed hepatic AVS. With an incidence of about 4.3%, hepatic AVS can be secondary to a variety of liver diseases. Generally, 89% of hepatic AVS patients have no obvious clinical symptoms, but in some hepatic AVS, especially hepatic arterial portal venous shunt, there are also existing liver function injury and simultaneously the formation of PH, which may also be related to ascites or GEVs.^[43,44]^ This PH caused by hepatic AVS is often difficult to treat effectively by routine symptomatic treatment, and interventional embolization is the only rapid and effective treatment at present, recommended as the first-line treatment in clinic.^[45]^ The diagnosis, location, partial flow, and vascular direction of hepatic AVS are determined by digital subtraction angiography through the celiac artery. In this procedure anhydrous ethanol is slowly perfused until the AVS disappears, and the total amount of ethanol is generally controlled to be below 10 mL. For patients with excessive partial flow, gelatin sponge particles or spring rings are used to reduce the shunt blood flow before the ethanol is injected. Hepatic AVS on the basis of HCC is quite different from the above. Here, patients may not only have liver function injury and PH but may also have a risk of cancer metastasis in the blood due to the existence of the shunt. In addition, the injection treatment of embolic agents and chemotherapeutic drugs may leak from the shunt, reducing the curative effect and increasing the risk of distant embolism. The standard treatment of HCC complicated with AVS is transcatheter arterial chemoembolization,^[46]^ and as briefly mentioned above the most commonly used materials for blocking AVS are spring coil, anhydrous ethanol, gelatin sponge, iodized oil, and microspheres. Treatment of HCC with AVS is not only conducive to transcatheter arterial chemoembolization treatment but can also can significantly reduce PP and improve liver function.^[47]^

The treatment for all the different etiologies of PH mentioned above are shown in Table 1.

**Table 1:** Treatment of different etiologies of PH.

Diseases	Etiology treatment	Medical treatment	Endoscopic therapy	Interventional therapy	Surgical treatment
Liver cirrhosis	Antiviral treatment in viral hepatitis	Without GEVs or with mild varicose veins: NSBBs are not recommended ② With moderate and severe varicose veins: NSBBs as primary prevention ③ VH: vasoactive drugs ④ Statins may reduce PP	① EVL, as primary prevention of GEVs ② VH: EVL, EIS ③ Gastric variceal bleeding: endoscopic tissue adhesive injection with or without EUS guided, as primary or secondary prevention	① When acute VH cannot be controlled, or at high-risk of treatment failure: TIPS, taken within 72 h, preferably within 24 h ② Gastric varices: BRTO ③ EUS-guided intrahepatic portosystemic shunt to treat PH	① Devascularization ② Surgical shunt ③ Liver transplantation
PPH	Treatment of primary pancreatic diseases		VH: EVL, EIS	① Partial embolization of the splenic artery ② Percutaneous splenic phleboplasty ③ TIPS seems to be ineffective	① Splenectomy: most effective method for PPH or later VH ② Shunt surgery, such as coronary-caval shunt
BCS		Complicated with venous thrombosis: thrombolytic treatment		① Membranous obstructive of hepatic vein or the IVC: balloon dilatation ② Segmental occlusion of hepatic vein or the IVC: implantation of intravascular stents after balloon dilatation ③ Due to extensive obstruction of hepatic veins and no recanalization conditions: TIPS ④ Due to abnormal and/or distorted anatomy, TIPS can be infeasible: DIPS or TTIPS	① Radical correction ② S hunt ③ Devascularization ④ Liver transplantation
INCPH		NSBB as primary prevention ② VH: vasoactive drugs are recommended ③ INCPH with PVT: anticoagulant drugs	EVL or EIS	TIPS	Liver transplantation
AVS				① Anhydrous ethanol interventional embolization ② HCC complicated with AVS: TACE	Liver transplantation

AVS = arteriovenous shunt, BCS = Budd-Chiari syndrome, BRTO = balloon-occluded retrograde transvenous obliteration, DIPS = direct intrahepatic portocaval shunt, EIS = endoscopic injection sclerosis, EUS = endoscopic ultrasound, EVL = endoscopic variceal ligation, GEVs = gastroesophageal varices, INCPH = idiopathic non-cirrhotic portal hypertension, IVC = inferior vena cava, NSBBs = non-selective β-blockers, PH = portal hypertension, PP = portal pressure, PPH = pancreatic portal hypertension, TACE = transcatheter arterial chemoembolization, TIPS = transjugular intrahepatic portosystemic shunts, TTIPS = transjugular transcaval intrahepatic portosystemic shunt, VH = variceal hemorrhage.

## 6. Conclusion

There are many causes of PH, but this paper only summarizes the common ones in clinic. The authors hope that physicians will deepen the differential diagnosis of the different causes and choose drugs, endoscopy, intervention, or surgical treatment on the basis of a full understanding of the PH in order to benefit patients to the greatest extent possible.

## Acknowledgments

The authors thank AiMi Academic Services (www.aimieditor.com) for the English language editing and review services.

## Author contributions

**Supervision:** Lan-qing Ma.

**Writing – original draft:** Ming-ke Li.

**Writing – review & editing:** Ming-ke Li, Lan-qing Ma.
